# Concealed malfunction of the temporary pacemaker

**Published:** 2006-04-01

**Authors:** Mohammad Alasti, Majid Haghjoo, Abolfath Alizadeh, Mohammad Ali Sadr-Ameli

**Affiliations:** Department of Pacemaker and Electrophysiology, Rajaie Cardiovascular Medical and Research Center, School of Medicine, Iran University of Medical Sciences, Tehran, Iran.

The 12-lead ECG shows sequential atrial and ventricular pacing (Figure 1A). A tracing, obtained simultaneously during pacemaker interrogation, disclosed pacemaker functioning as VDD mode (Figure 1B). The careful examination of this pacemaker tracing showed that there is a pacing stimulus before each P wave (compatible with DDD mode). This paradox can only be explained by displacement of the temporary pacing lead to right atrium and right atrial stimulation by temporary pacemaker. In this setting, each temporary pacemaker-induced atrial depolarization is tracked by the right atrial lead of the permanent pacemaker as intrinsic P wave. Fluoroscopic study confirmed this explanation (Figure 2). The displaced temporary pacing lead was seen near the lateral right atrial wall. Temporary pacemaker lead had been inserted before replacement of permanent pacemaker.

## Figures and Tables

**Figure 1:**
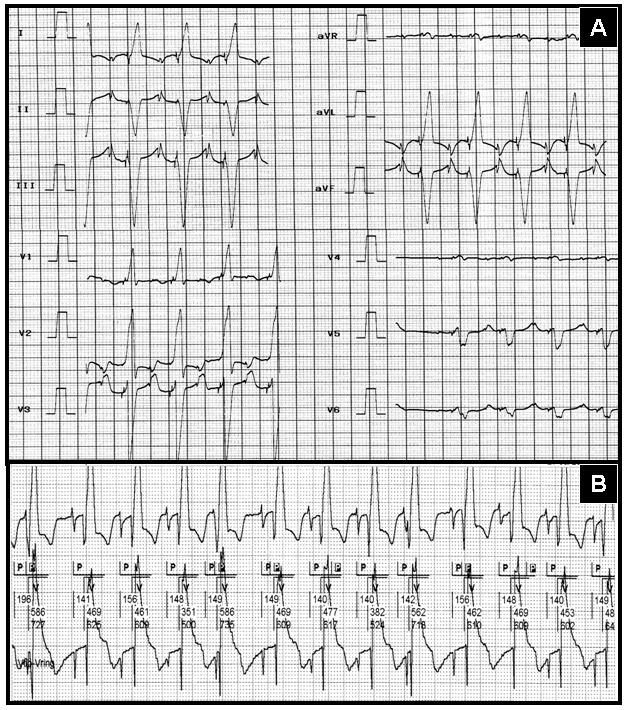
**A**, 12-lead electrocardiogram showing sequential atrial and ventricular pacing. **B**, pacemaker tracing obtained simultaneously is compatible with VDD mode.

**Figure 2:**
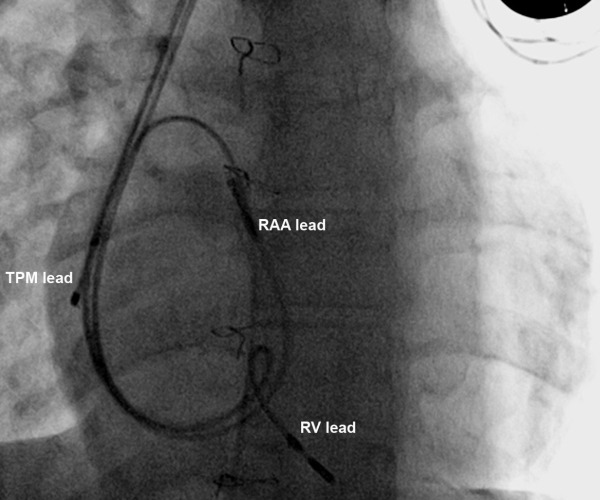
AP fluoroscopic view shows atrial and ventricular leads of the permanent pacemaker and the tip of dislodged temporary pacemaker in the lateral wall of right atrium.

